# Pineal parenchymal tumors of intermediate differentiation: in need of a stringent definition to avoid confusion. Scientific commentary on ‘Genetical and epigenetical profiling identifies two subgroups of pineal parenchymal tumors of intermediate differentiation (PPTID) with distinct molecular, histological and clinical characteristics’

**DOI:** 10.1007/s00401-024-02684-3

**Published:** 2024-02-10

**Authors:** Alexandre Vasiljevic

**Affiliations:** 1 Department of Pathology and Neuropathology, Hospices Civils de Lyon, Bron, France; 2 Faculty of Medicine Lyon Est, Claude Bernard Lyon 1 University, Lyon, France

In their recent publication in Acta Neuropathologica, Rahmanzade, et al., described a new subgroup of pineal parenchymal tumor of intermediate differentiation (PPTID), suggested to be referred as ‘PPTID-C’ [8]. Their interesting study raises a very critical issue regarding the terminology of pineal parenchymal tumors (PPTs): what neoplasm should be named PPTID?

PPTIDs have been first included in the 2000 WHO classification (3rd edition) in which they were defined as tumors composed of diffuse sheets of monomorphous cells without large pineocytomatous pseudorosettes [3]. Their name implies that they are ‘intermediate’ between pineocytoma (PC) and pineoblastoma (PB) in terms of cellularity, nuclear atypia, mitotic activity, and prognosis. They partly replaced the concept of mixed PC-PB that was controversial as some cases corresponded to PB invading normal pineal gland. In the next editions of the WHO classification (2007 (4th edition) and 2016 (revised 4th edition)), the histopathological description of PPTID was enriched [6, 7]. Besides the ‘classic’ diffuse architecture, a lobulated, transitional, and pleomorphic variants were introduced. The ‘lobulated’ and ‘transitional’ architectures of PPTID were coined in a study by Jouvet, et al., in 2000 [2]. The ‘transitional’ cases were defined as PPTs showing typical PC-like areas with pineocytomatous pseudorosettes ‘in transition’ with diffuse sheets of neoplastic cells that are more reminiscent of PPTID. In this publication, the authors also suggested a grading system for PPTID, separating low-grade tumors (grade 2) from high-grade tumors (grade 3) using mitotic count and Neurofilament protein (NFP) immunohistochemistry [2]. Using this grading, ‘transitional’ PPTID were classified as grade 2 tumors. In addition, pleomorphic cells, a feature typical of PC, have been reported in Jouvet’s grade 2 PPTID by the same authors, hence the mention of the ‘pleomorphic’ variant of PPTID in WHO classifications [1].

According to the 2021 WHO classification of central nervous system tumors (5th edition), PPTID is defined as a neoplasm with diffuse or vaguely lobulated architecture that morphologically may resemble central neurocytoma or oligodendroglioma [9]. They lack the large pineocytomatous pseudorosettes that are typical of PC. They show diffuse synaptophysin immunoexpression and scarse NFP cytoplasmic immunopostivity. For the first time, a molecular alteration has been included as a desirable criterion for PPTID’s diagnosis. Among PPTs, small in-frame insertion in the *KBTBD4* gene (coding for a protein involved in ubiquitination) is characteristic of PPTID as it is found in up to 80% of cases and not in PC or PB [4, 5, 10]. As such, *KBTBD4*-mutated PPTID appears to be the prototypical PPTID. The prognosis of these tumors and their grading is still debated (grade 2 or 3) and no definite grading criteria have been validated. In the new 2021 WHO classification, the notion of ‘transitional’ and ‘pleomorphic’ variants of PPTID have not been maintained. Indeed, it appears that these cases diverge from the *KBTBD4*-mutated PPTID and that they more accurately belong to the PC family.

**Fig. 1:**
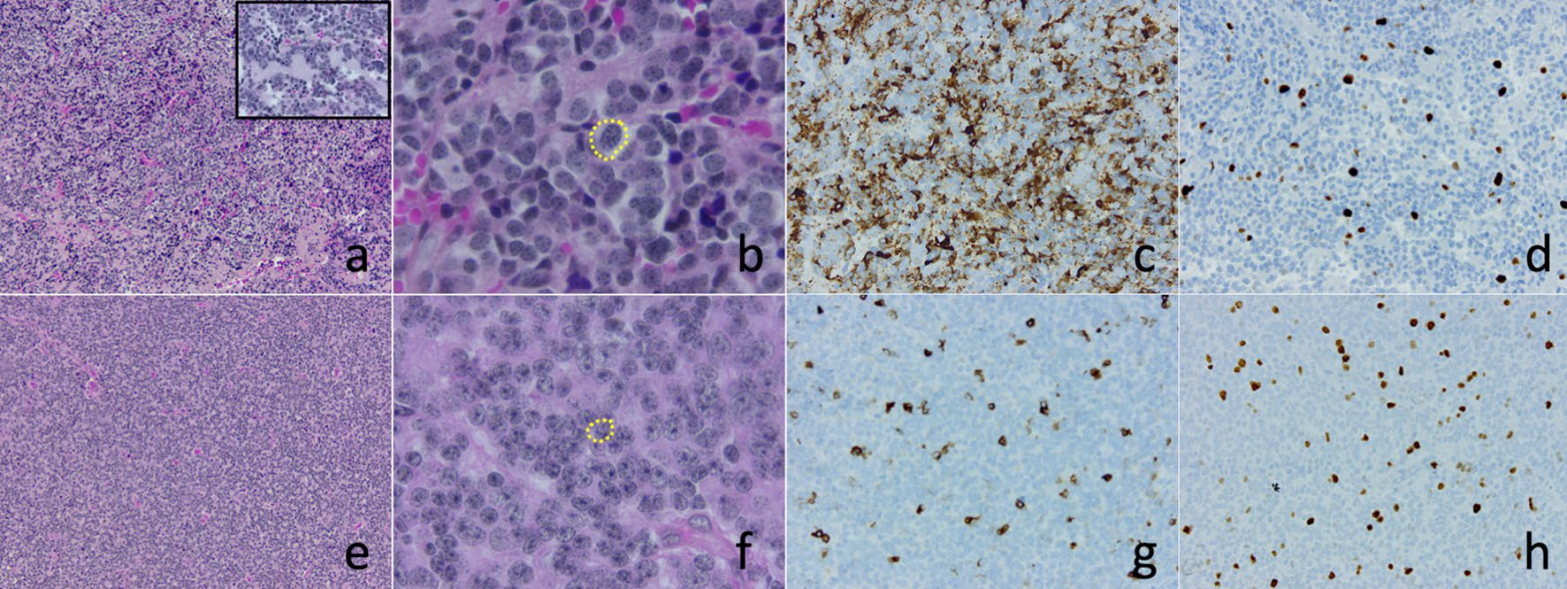
Differences between PPTID-C and *KBTBD4*-mutated PPTID: The pineal tumor depicted in pictures **a** to **d** shows features consistent with the diagnosis of pineal parenchymal tumor of intermediate differentiation (PPTID)-C. In contrast to *KBTBD4*-mutated PPTID (**e**, Hematoxylin, Phloxine, Saffron (HPS) staining, × 100), this tumor shows rare foci of pineocytomatous pseudorosettes (transitional architecture) (**a**, HPS × 100 and insert, HPS × 600). Molecularly, PPTID-C is *KBTBD4*-wild type and shows 13q loss. Its methylation profile matches with pineocytoma (score = 0.99 in v12.5 of the Heildelberg classifier; www.molecularneuropathology.org). Compared with *KBTBD4*-mutated PPTID (**e**–**h**), PPTID-C (**a**–**d**) are composed of larger cells (**b** > **f**, HPS × 600) and show higher Neurofilament Protein immunopositivity (**c** > **g**, HPS × 200). The Ki67 hotspot proliferation index is here in the range of Rahmanzade’s report (**d** = 6.3% and **h** = 8.3%) with no significant difference

Taking into consideration these earlier definitions (‘transitional’ and ‘pleomorphic’ variants of PPTID) together with the more classic definition of PPTID, Rahmanzade, et al., described a new subgroup of PPTID [8]. This new subgroup, named ‘PPTID-C’, is mainly characterized by transitional morphology (70%) with large cells, significant NFP immunopositivity, absence of *KBTBD4* alteration, and loss of chromosome 13q (60%) (Table 1). By methylation analysis, they cluster with PC and significantly differ from the PPTID A and B methylation classes. When compared with classic *KBTBD4*-mutated PPTID, their prognosis is better. Integrating all these data, it appears that PPTID-C, similar to neoplasms referred as ‘transitional’ PPTID, ‘pleomorphic’ PPTID, Jouvet’s grade 2 PPTID, mostly correspond to PC and differ from the *KBTBD4*-mutated prototypical PPTID (Fig. 1). The inclusion of PPTID-C in the family of PC is consistent with their histopathological features, methylation profile, and favorable clinical behavior (Table 1). The counterpart of this statement is that the PC group is heterogeneous. It remains thus to clarify the morphological spectrum of PC and the impact of 13q loss in their prognosis as histologically typical PC (*ie* with pineocytomatous pseudorosettes and not PPTID-like diffuse areas) are less likely to harbor this chromosomal imbalance according to the study of Rahmanzade, et al.[8]. In line with this, grade 1 typical PC with large pseudorosettes show a better prognosis than Jouvet’s grade 2 PPTID [2].

**Table 1:** Comparison between Pineocytoma, Pineal parenchymal tumor of intermediate differentiation group C (PPTID-C) and PPTID as defined by the 2021 WHO classification

	Pineocytoma	‘PPTID-C’	PPTID
CNS WHO grade	Grade 1	NA	Grade 2/3
Jouvet’s grading system	PC grade 1	PPTID grade 2	PPTID grade 2/3
Main morphological features	Pineocytomatous pseudorosettes; no PPTID-like diffuse areas	Transitional architecture	Diffuse/Vaguely lobulated Central neurocytoma-like (+ + +) or oligodendroglioma-like
Pseudorosettes	**Pineocytomatous**	**Pineocytomatous** *(focal in transitional variant)*	Small pseudorosettes possible
Cell size	**Larger than in PPTID with *KBTBD4* mutation**	**Larger than in PPTID with *KBTBD4* mutation**	Smaller than in ‘PPTID-C’ and PC
Pleomorphic cells	**Possible**	**Possible**	No
NFP immunoexpression	** + + **	** + + **	Scant
*KBTBD4* mutation	**Negative**	**Negative**	Positive (80%)
13q loss	12.5%	60%	13%
Methylation profile	**PC**	**PC**	PPTID
Prognosis	**Favorable**	**Favorable**	Intermediate

*CNS* central nervous system, *NA* not applicable, *NFP* neurofilament protein, *PC* pineocytoma, *PPTID* pineal parenchymal tumor of intermediate differentiation, *WHO* World Health Organization

Before the molecular area, the misdiagnosis of some PC and PB as PPTID (respectively as grade 2 and 3 PPTID) has mechanically led to some misinterpretation of the prognosis and therapeutic management of PPTID. A clear definition of PPTID is thus needed to avoid any confusion between PPTs and to allow reliable clinical studies with homogeneously-defined PPTID.

In conclusion, we suggest that the group of pineal tumors defined by Rahmanzade, et al., as PPTID-C, more accurately correspond to a subgroup of PC. This emphasizes the need of a stringent definition of PPTID. In this setting, the role of *KBTBD4* status appears critical. It remains now to better define the morphological and behavioral spectrum of PC for which we still lack a ‘signature’ molecular alteration, and to see if the *KBTBD4* alteration should be endorsed as a defining feature of PPTID. For the future WHO classification, we suggest the following definition of PPTID for discussion: ‘a *KBTBD4*-mutated pineal parenchymal tumor characterized by diffuse sheets of monomorphous round cells, lacking pleomorphism and large irregular pseudorosettes, and presenting limited NFP immunopositivity and moderate proliferative activity’.
